# Supplementary material for: Lifestyle Modification for Enhancing Autonomic Cardiac Regulation in Children: The Role of Exercise
Source: Children (Basel). 2019 Nov 16;6(11):127. doi: 10.3390/children6110127 (PMC6915468; doi:10.3390/children6110127)
Supplement: Supplementary file 1 [file children-06-00127-s001.pdf]

Table S1: Summary of Accepted Articles.

Author (Date)	Participants	Aim	Exercise Sub-Topic	Findings
van Biljon et al. (2018)	-*n* = 109 (M and F)<br>-Age: 10 – 13 years old<br>-Condition: healthy	To investigate the impact of 3 isocaloric exercise programs on cardiac ANS functioning in children	-Mode: HITT, MICT and ALT<br>-HITT duration: 23-min<br>-MICT duration: 33-min<br>-Frequency: 3 days/week<br>-Intensity: moderate and high intensity<br>-Intervention length: 5 weeks	-HITT resulted in significant enhancements in HRV compared with MICT and ALT<br>-Findings suggest that both magnitude and the components of the ANS relate to exercise intensity
Cohen-Holzer et al. (2017)	-*n* = 24 (M and F)<br>-Age: 6 – 10 years old<br>-Condition: UCP	To examine the influence of an intensive hybrid program on cardiac autonomic regulation, walking endurance and the correlation with upper extremity function of children with UCP	-Mode: aerobic<br>-Duration: 6 hours/day<br>-Frequency: 5 days/week<br>-Intervention length: 2 weeks	A significant increase in HRV (RMSSD) at post- and 3-months post-intervention was noted as well as walking improvement and arm function
Sharma et al. (2017)	-*n* = 439 (M and F)<br>-Age: 12 – 17 years old<br>-Condition: healthy	To compare the effect of structured and unstructured exercise on maximal aerobic capacity and HRV among adolescents	-Mode: structured (muscle and bone strengthening exercise and stretching) vs unstructured PA<br>-Duration: 2 hours/day<br>-Frequency: 6 days/week<br>-Intensity: vigorous<br>-Intervention length: 6 months	HRV increased significantly from pre- to post-testing for both interventions however structured PA was more beneficial for improving HRV than unstructured PA irrespective of gender and sports activities
Farinatti et al. (2016)	-*n* = 44 (M and F)<br>-Age: 13 – 17 years<br>-Condition: obese	To investigate the effects of RT on HRV in obese teens	-Mode: RT circuit<br>-Duration: 30-40-min<br>-Frequency: 3 days/week<br>-Intervention length: 12 weeks	-RT attenuated ANS dysfunction in obese adolescents by increasing PNS activity and decreasing sympathovagal balance<br>-PNS indices increased and baseline differences between obese and

				healthy groups were no longer observed after RT
Bond et al. (2015)	-*n* = 13 (M and F)<br>-Age: 13 – 14 years old<br>-Condition: healthy	To identify the influence of of HITT on CVD risk factors in fasted and postprandial states in adolescents	- Mode: aerobic (HITT cycling)<br>-Intervention length: 2 weeks	-HITT improved HRV in adolescents<br>-Most improvements in HRV were lost 3 days after HITT
Shin et al. (2014)	-*n* = 15 (M only)<br>-Age: ~13 years old<br>-Condition: type 1 diabetes	To investigate the effect of exercise on ANS CV risk profiles in children with type 1 diabetes	-Mode: aerobic (walking)<br>-Frequency: 3x/week<br>-Intensity: moderate<br>-Intervention length: 12 weeks	TP, LF and VLF significantly increased after exercise training
Satish et al. (2013)	-*n* = 24 (M and F)<br>-Age: 11 – 13 years old<br>-Condition: healthy	To evaluate the effect of yoga on HRV as a stress index in sub-junior cyclists	-Mode: yoga<br>-Duration: 1 hour<br>-Frequency: 7 days/week<br>-Intervention length: 4 weeks	-Yoga significantly decreased LF and LF/HF and increased HF<br>-HF significantly decreased in the control group
Prado et al. (2010)	-*n* = 33 (M and F)<br>-Age: 10 years old<br>-Condition: obese	To test the hypothesis that in obese children: 1) hypocaloric diet improves HRR and cardiac ANS activity and 2) diet and exercise combined leads to greater improvement in HRR and ANS activity	-Mode: aerobic<br>-Duration: 1 hour<br>-Frequency: 3 days/week<br>-Intervention length: 4 months	-Obese children in the diet and exercise group had greater improvements in ANS activity<br>-Obese children in the diet only group showed no difference in ANS activity after the intervention
Gamelin et al. (2009)	-*n* = 38 (M and F)<br>-Age: ~ 9 years old<br>-Condition: healthy	To observe the effect of high intermittent exercise training on children's HRV	-Mode: aerobic (running)<br>-Duration: 30-min<br>-Frequency: 3 days/week<br>-Intensity: maximal and supramaximal<br>-Intervention length: 7 weeks	No significant differences were found in HRV between the exercise intervention and control groups

Nagai et al. (2004)	-n = 305 (M and F)<br>-Age: 6 – 11 years old<br>-Condition: healthy	To investigate the effect of a long-term moderate exercise program on ANS activity in healthy children	-Mode: aerobic<br>-Duration: 20-min<br>-Frequency: 5 days/week<br>-Intensity: moderate<br>-Intervention length: 12 months	-12 months of short duration moderate exercise training has a positive effect on ANS activity in children who initially had a low HRV<br>-In the low HRV group, all HRV indices significantly increased after training
Mandigout et al. (2002)	-n = 19 (M and F)<br>-Age: 10 – 11 years old<br>-Condition: healthy	To evaluate the effect of an endurance training program on HRV in prepubertal healthy children	-Mode: aerobic<br>-Duration: 1 hour<br>-Frequency: 3 days/week<br>-Intensity: vigorous<br>-Intervention length: 13 weeks	Exercise significantly increased global HRV as compared to the controls
Gutin et al. (2000)	-n = 79 (M and F)<br>-Age: 7 – 11 years old<br>-Condition: obese	To investigate changes over an 8-month period in 2 groups of obese children	-Mode: aerobic<br>-Duration: 40-min<br>-Frequency: 5 days/week<br>-Intensity: moderate to vigorous<br>-Intervention length: 4 months	-Regular exercise had a favourable effect on PNS activity in obese children<br>-RMSSD increased in the 4-month period during which children received the intervention and then declined in the 4-month period following cessation of the intervention
Gutin et al. (1997)	-n = 35 (M and F)<br>-Age: 7 – 11 years old<br>-Condition: obese	To investigate whether physical training would shift the balance of ANS activity in obese children	-Mode: aerobic<br>-Duration: 40-min<br>-Frequency: 5 days/week<br>-Intensity: moderate to vigorous<br>-Intervention length: 4 months	Compared to the control group, the trained group demonstrated increased RMMSD, decreased LF and decreased LF/HF

Note: **M**, Males; **F**, Females; **ANS**, autonomic nervous system; **HRV**, heart rate variability; **PNS**, parasympathetic nervous system; **CVD**, cardiovascular disease; **CV**, cardiovascular; **HITT**, high intensity interval training; **MICT**, moderate intensity interval training; **ACT**, alternate HITT and MICT; **PA**, physical activity; **HRR**, heart rate recovery; **UCP**, unilateral cerebral palsy; **RT**, resistance training; **SDNN**, standard deviation of normal-to-normal interbeat intervals; **RMSSD**, root mean square of successive differences; **TP**, total power; **VLF**, very low frequency; **LF**, low frequency; **HF**, high frequency; **LF/HF**, the ratio between low and high frequency
